# Programmed death ligand 1 and tumor-infiltrating CD8^+^ T lymphocytes are associated with the clinical features in meningioma

**DOI:** 10.1186/s12885-022-10249-4

**Published:** 2022-11-12

**Authors:** Yaochuan Zhang, Xiaoliang Wang, Mengwu Shi, Yifu Song, Juanhan Yu, Sheng Han

**Affiliations:** 1 Department of Neurosurgery, The First Hospital of China Medical University, Shenyang, 110001 China; 2 Department of Pathology, First Affiliated Hospital and College of Basic Medical Sciences, China Medical University, Shenyang, 110001 China

**Keywords:** Meningioma, PD-L1, CD8^+^ TILs, Tumor environment, Immunotherapy

## Abstract

**Objective:**

To investigate the expression of programmed death ligand-1 (PD-L1) and the levels of CD8^+^ tumor-infiltrating lymphocytes (TILs) in meningioma as well as determine the association between their levels and the clinical outcomes.

**Methods:**

We performed a retrospective case-control study on 93 patients with meningioma. The patients showed tumor recurrence and were matched with the control patients without recurrence in their age, gender, admission time, tumor sites, tumor volume, peritumoral brain edema (PTBE), Simpson grade resection, WHO grade, postoperative radiotherapy, and the follow-up duration. We reviewed the clinical data of patients and performed immunohistochemistry analysis to investigate the PD-L1 expression and the levels of CD8^+^ TILs. Multivariate logistic regression was performed to analyze the association between clinical features and immune markers. The conditional logistic regression models were used to calculate the odds ratios (ORs) with 95% confidence intervals (CIs), and Kaplan–Meier analysis was performed to analyze tumor recurrence.

**Results:**

Tumor volume was correlated with the PD-L1 expression (*P* = 0.003, HR = 5.288, 95%CI, 1.786–15.651). PTBE served as an independent predictor of CD8^+^ TIL levels (*P* = 0.001, HR = 0.176, 95%CI 0.065–0.477). The levels of CD8^+^ TILs were associated with tumor recurrence (*P* = 0.020, OR = 0.325, 95%CI, 0.125–0.840).

**Conclusion:**

Tumor volume was associated with PD-L1 expression, and PTBE was an independent predictor of CD8^+^ TIL levels in meningioma. CD8^+^ TIL levels correlated with tumor recurrence in meningioma.

**Supplementary Information:**

The online version contains supplementary material available at 10.1186/s12885-022-10249-4.

## Introduction

Meningioma is one of the most common primary intracranial tumors. Most meningiomas are benign [1]. The main treatments for meningioma include surgery, radiotherapy, and radiosurgery [2]. Even though a majority of patients can be treated with surgery, few patients with malignant, refractory, or recurrent meningioma do not respond well to treatments. The gross total resection of meningiomas located in the cavernous sinus and petroclival region is difficult owing to a high risk of severe complications [1, 2]. Moreover, a consensus remains to be established about the efficacy of radiation therapy for such meningiomas [2]. Therefore, new treatments are urgently required for these patients, and immunotherapy can be a promising treatment strategy.

Tumor cells express programmed death ligand 1 (PD-L1), which interacts with the activated T cells that show high expression of programmed cell death 1 (PD-1). They may be involved in tumor escape and promotion of tumor growth, proliferation, and survival by inducing T lymphocyte apoptosis, suppressing T lymphocyte toxicity, and promoting the differentiation of cytotoxic T lymphocytes into regulatory T cells [3, 4]. Thus, blocking this axis can reverse the inhibition of antitumor immune responses and reactivate the T cells to kill tumor cells [5, 6]. Accordingly, anti-PD-1/PD-L1 immunotherapy has shown clinical benefits in different tumor types, such as non-small-cell lung cancer (NSCLC) [7], ovarian cancer [8], melanoma [9], and bladder cancer [10]. Therefore, PD1/PD-L1 immunotherapy can be a promising treatment strategy for recurrent meningioma. Many clinical trials are ongoing, such as NCT03016091, NCT03279692, NCT02648997, NCT03604978, and NCT03267836. One of these trials had shown that pembrolizumab, a PD-1 inhibitor, gives favorable efficacy on a subset of recurrent and higher-grade meningiomas [11]. An in-depth understanding of the tumor microenvironment (TME) in meningioma is required to design an optimal PD1/PD-L1 immunotherapy. However, only a few studies have investigated TME in meningioma to date.

CD8^+^ tumor-infiltrating lymphocytes (CD8^+^ TILs) are important cells of the immune system that exert antitumor effects. Therefore, they play a crucial role in TME [12]. The loss of function or dysfunction of CD8^+^ TILs affects NSCLC progression [13]. Low CD8^+^ TIL levels are associated with poor prognosis in glioma [14, 15]. In addition, the presence of pre-existing antitumor T cells, particularly CD8^+^ TILs, is a considerable predictor of the response of patients to PD-1/PD-L1 immunotherapy [16]. However, limited studies have investigated the role of CD8^+^ TILs and PD-L1 individually or in combination in meningioma to date. In this retrospective study, we reviewed the cases of 93 patients with meningioma and performed immunohistochemistry (IHC) analysis to investigate the expression of PD-L1 and the levels of CD8^+^ TILs and determined the association between their levels and clinical outcomes.

## Methods

### Study patients

Between January 2011 and February 2021, a total of 3523 consecutive patients were diagnosed with meningioma by 2 neuropathologists at the First Hospital of China Medical University, and the presence of other tumors was simultaneously excluded. From these patients, we selected 1022 patients with primary meningioma and with complete clinical data, formalin-fixed paraffin-embedded tissues for IHC analysis, and clinical follow-up information. To explore the association between the immune markers and tumor recurrence, we included all patients with tumor recurrence in the study. These cases were matched with the corresponding controls for the confounding factors, which included age, gender, admission time, tumor sites, tumor volume, peritumoral brain edema (PTBE), Simpson grade resection, WHO grade, postoperative radiotherapy, and the follow-up duration. Finally, 31 cases were successfully matched with 62 controls. We then collected the patients’ clinical data through the electronic medical record system of the First Hospital of China Medical University. Based on the data obtained from preoperative magnetic resonance imaging (MRI), the tumor volume was calculated using the following formula: ∑ (anteroposterior diameter × lateral diameter × axial diameter) × π/6. The results obtained with this calculation and the intraoperative measurements were mutually verified. PTBE and postoperative brain edema were examined by MRI. The same surgical methods and principles were employed for tumor resection. The WHO grade was defined in accordance with the 2016 WHO criteria. The definition of the Simpson grade has been summarized in Supplementary Table 1. Recurrence-free survival (RFS) was defined as the period between surgery and meningioma recurrence. The institutional review board of the First Hospital of China Medical University approved this study protocol (Approval No. 2022098), and we obtained written informed consent from all meningioma tissue donors who agreed to the use of their tumor tissues and clinical data for future research.

### IHC and immunofluorescence analyses

IHC staining was performed as described previously [15]. Briefly, paraffin-embedded meningioma tissues were cut into 4-μm-thick slices and deparaffinized with xylene. Then, the antigen was retrieved at high temperature and pressure in sodium citrate buffer (pH 6.0). IHC analysis was then performed by using the UltraSensitive™ S-P kit (KIT-9720; Maixin Biotech, Fuzhou, China) in accordance with the manufacturer’s instructions. Briefly, after blocking with endogenous peroxidase and nonspecific staining, the sections were incubated with primary antibodies against CD8 (Cat No. 66868–1-Ig, 1:4000, Proteintech, Wuhan, China), PD-L1 (Cat No. 66248–1-Ig, 1:500, Proteintech), VEGF (Cat No. 19003–1-AP, 1:100, Proteintech), and CD163 (Cat No. 16646–1-AP, 1:100, Proteintech) proteins. Normal mouse serum was used to replace the primary antibody in the negative control, and the tonsil section was stained and used as the positive control. Then, the sections were incubated with a biotin-labeled secondary antibody to amplify the signal. The secondary antibody was conjugated with streptavidin-peroxidase, and the sections were stained with diaminobenzidine and then counterstained with hematoxylin. For double-labelled immunofluorescence staining, PD-L1 and CD163 were detected with tetramethylrhodamine isothiocyanate-conjugated anti-mouse immunoglobulin G (IgG) (Cat No. SA00007–1, 1:100, Proteintech) and fluorescein isothiocyanate (FITC)-conjugated anti-rabbit IgG (Cat No. SA00003–2, 1:100, Proteintech), while the nuclei were counterstained with DAPI (Fluoroshield with DAPI, F6057; Sigma). The immunofluorescence sections were imaged using a fluorescence microscope (Olympus, Tokyo, Japan). The light microscope used was connected to a computer (Olympus), and the IHC results were photographed. The presence of a brownish-yellow patchy pattern on the cell membrane and the cytoplasm was indicative of a positive result for the PD-L1 protein. The presence of brownish-yellow staining on the cell membrane was indicative of a positive result for CD8^+^ TIL. VEGF protein was mainly expressed in the cytoplasm. Positive results were confirmed by 2 neuropathologists. To determine the PD-L1 and VEGF protein expression, each section was analyzed from at least 5 randomly selected areas at high-power fields (HPFs, 400×) with reference to the average optical density (integrated optical density/area) calculated with the Image-Pro Plus 6.0 (IPP; Media Cybernetics, Inc. Silver Spring, MD, USA). For CD8^+^ TILs, each section was evaluated in 5 different HPFs (400×) with the richest levels of CD8^+^ TILs. The enumeration was repeated at least thrice independently by 2 expert neuropathologists who were blinded to the clinical information. To ensure repeatability, all the results were rechecked after a predefined period. If the results were consistent, the average value of each section was used in the next statistical analyses. All photos were captured under the same exposure and white balance conditions.

### Statistical analyses

Chi-square test and Student’s *t-*test were performed to assess the statistical significance. Univariate and multivariate logistic regression analyses were performed to analyze the factors affecting the PD-L1 expression and the levels of CD8^+^ TILs. Conditional logistic regression models were employed to calculate the odds ratios (ORs) with 95% confidence intervals (CIs), as well as to determine the association among the levels of CD8^+^ TILs and the PD-L1 expression and tumor recurrence, adjusted for age, gender, admission time, tumor sites, tumor volume, PTBE, Simpson grade resection, WHO grade, postoperative radiotherapy, and the follow-up duration. Kaplan–Meier survival curves were constructed to determine the distribution of RFS in accordance with the CD8^+^ TIL levels, while the PD-L1 expression was analyzed by the log-rank test. Cox proportional hazard models were also applied to assess the survival function of CD8^+^ TILs and PD-L1. SPSS v25.0 (SPSS Inc., Chicago, IL) and GraphPad Prism 7 (GraphPad Software Inc., La Jolla, CA) was exerted to perform all statistical analyses. *P* < 0.05 (two-tailed) was considered to indicate statistical significance.

## Results

### Clinical features

The clinical features of 93 patients with meningioma are summarized in Table 1. The cohort included 21 male patients (22.6%) and 72 female patients (77.4%); their average age was 46.28 ± 16.33 years (21–79 years). A total of 13 patients (14.0%) smoked and 17 patients (18.3%) consumed alcohol. The most common symptoms were visual impairment (*n* = 19, 20.4%), headache (*n* = 17, 18.3%), and limb weakness (*n* = 17, 18.3%). The average tumor volume was 47.79 ± 38.56 cm^3^ (1.46–252 cm^3^). A total of 36 patients (38.7%) had PTBE. Most of the tumors were located at the base of the skull (*n* = 66, 71.0%). Simpson grade I, II, III, or IV resection was achieved in 27 (29.0%), 21 (22.6%), 33 (35.5%), and 12 (12.9%) patients, respectively. The condition of 39 patients (42.0%) was diagnosed as higher-grade (WHO grade 2 and 3) meningioma, and 15 patients (16.1%) underwent postoperative radiotherapy.

**Table 1:** Clinical features of the 93 meningioma patients

Clinical features	No.	%
Total	93	100
Age (years)		
Mean ± SD	46.28 ± 16.33	
Gender		
Male	21	22.6
Female	72	77.4
Smoking		
No	80	86.0
Yes	13	14.0
Drinking		
No	76	81.7
Yes	17	18.3
Preoperative symptoms		
Visual impairment	19	20.4
Headache	17	18.3
Limb weakness	17	18.3
Epilepsy	9	9.7
Tumor volume (cm^3^)		
Mean ± SD	47.79 ± 38.56	
Peritumoral brain edema		
No	57	61.3
Yes	36	38.7
Tumor sites		
Convexity	15	16.1
Skull base	66	71.0
Ventricle	12	12.9
Simpson grade		
I	27	29.0
II	21	22.6
III	33	35.5
IV	12	12.9
WHO grade		
I	54	58.0
II	21	22.6
III	18	19.4
Postoperative brain edema		
No	78	83.9
Yes	15	16.1
Postoperative limb weakness		
No	87	93.5
Yes	6	6.5
Postoperative radiotherapy		
No	78	83.9
Yes	15	16.1

### PD-L1 and CD8^+^ TILs in meningioma

**Fig. 1:**
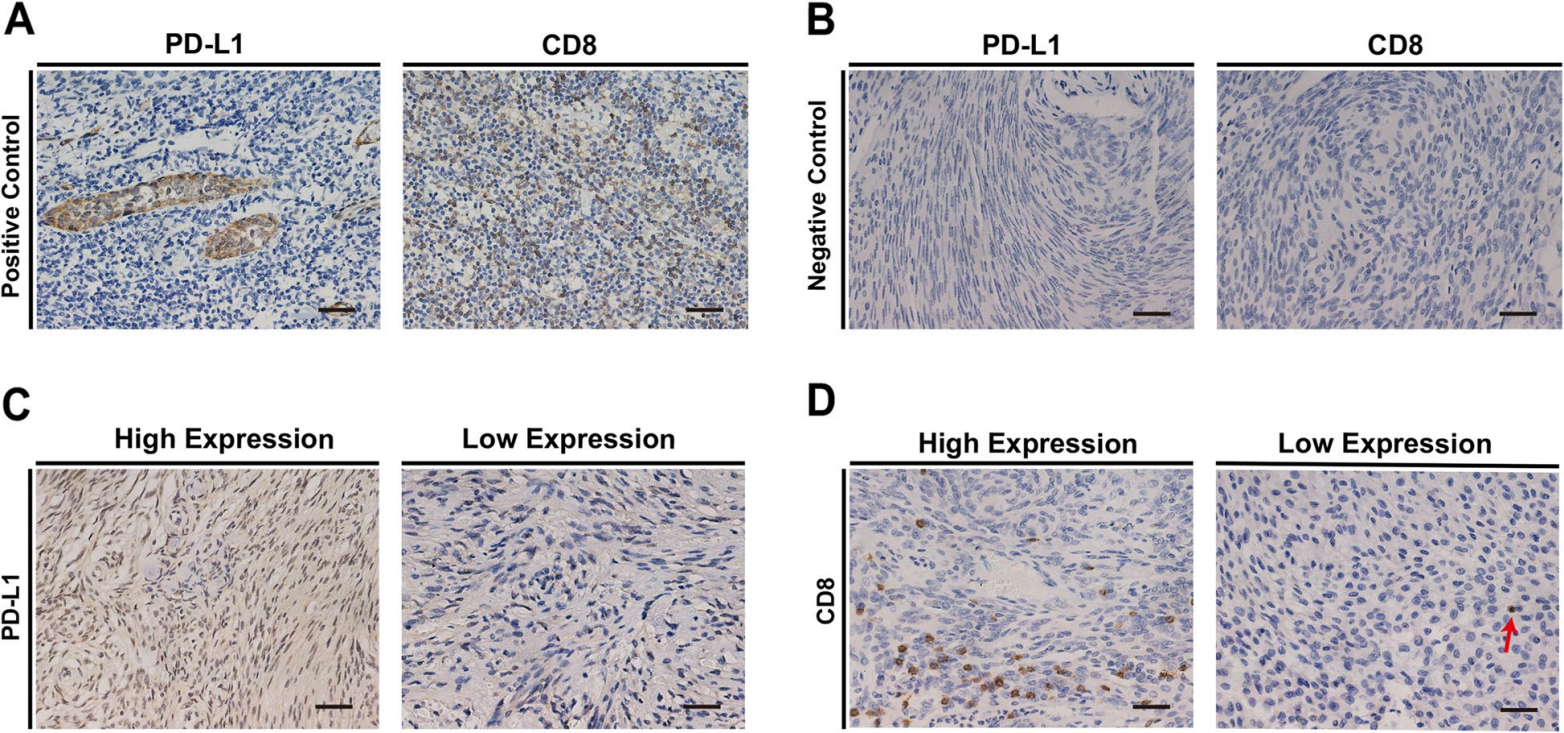
Representative images of immunohistochemical analysis in meningiomas. Positive control (**a**), negative control (**b**), PD-L1 protein (**c**), and CD8 protein (**d**) in immunohistochemistry analyses of meningiomas. Scale bar = 20 μm. PD-L1, programmed death ligand 1

Positive and negative controls were established in IHC analysis to eliminate false negative and positive results, respectively (Fig. 1a and b). The PD-L1 protein in the meningioma was distributed in a patch, both in the cell membrane and cytoplasm (Fig. 1c). CD8^+^ TILs were aggregated or scattered in the meningioma, and the CD8 protein was present in the cell membrane (Fig. 1d).

### PD-L1 expression, CD8^+^ TIL levels, and clinical features

**Fig. 2:**
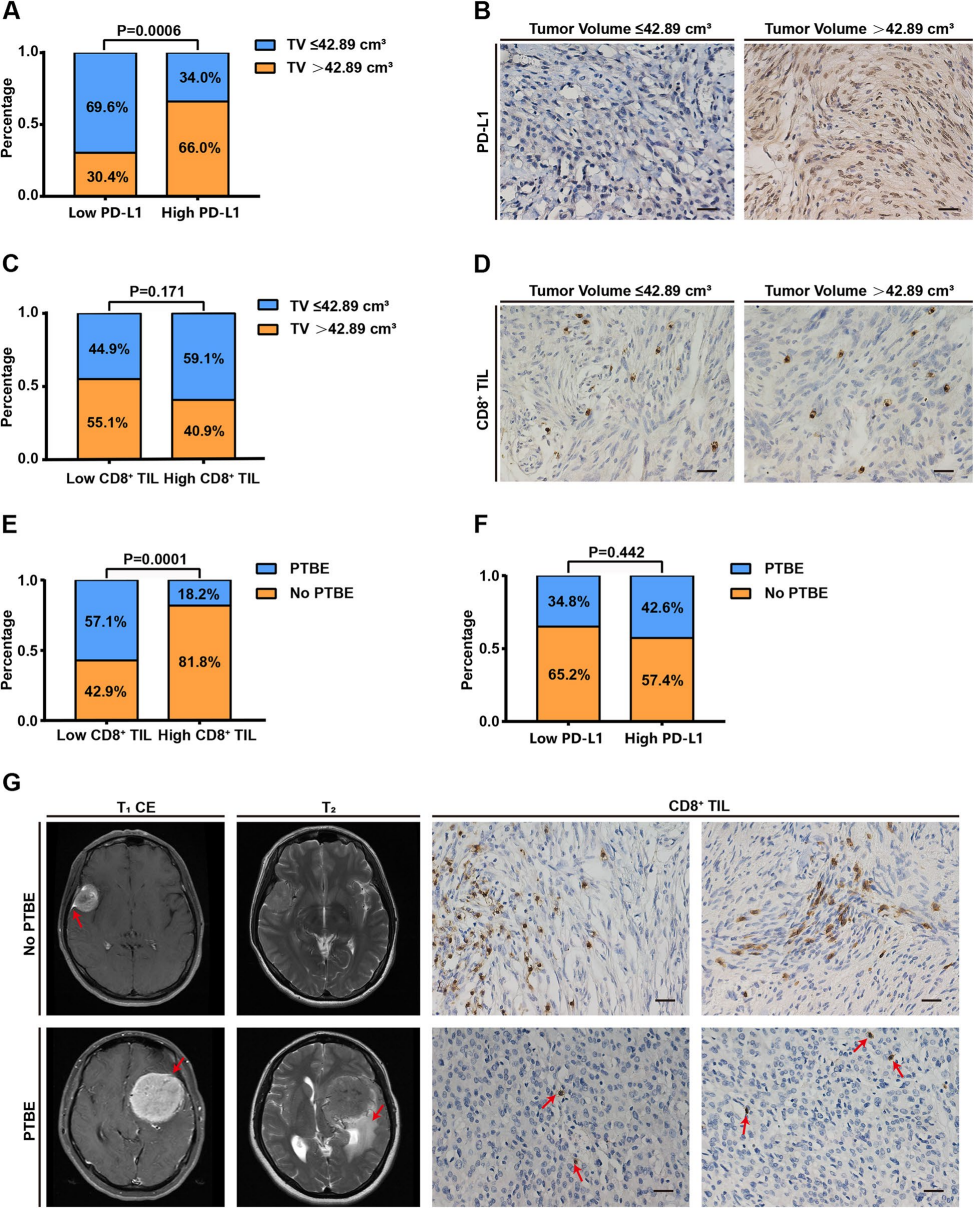
The association among the PD-L1 expression, CD8^+^ TIL levels, and clinical features. **a** The PD-L1 expression was significantly correlated with the tumor volume. **b** Cases with larger tumor volume indicated higher PD-L1 expression in IHC staining relative to those with a smaller volume. Scale bar = 20 μm. **c** The CD8^+^ TIL levels were not associated with the tumor volume. **d** The number of CD8^+^ TILs in IHC staining was not significantly different between the samples with small and large tumor volumes. Scale bar = 20 μm. **e** The CD8^+^ TIL levels were significantly associated with PTBE. **f** The PD-L1 expression was not associated with PTBE. **g** The number of CD8^+^ TILs in IHC staining was significantly higher in cases without PTBE than in those with PTBE. The red arrows in MRI images indicated a meningeal tail sign and PTBE, respectively. Scale bar = 20 μm. IHC, immunohistochemistry; PD-L1, programmed death ligand 1; TILs, tumor-infiltrating lymphocytes; TV, tumor volume; T_1_CE, T_1_WI contrast-enhancement; T_2_, T_2_WI; PTBE, peritumoral brain edema

We determined the association between the immune markers and the clinical features (Table 2). The median value of the PD-L1 expression was used as a cutoff point to divide the cohort into groups with low and high PD-L1 expression and similarly low and high CD8^+^ TIL levels. Patients with low PD-L1 expression had a significantly smaller tumor volume than those with higher PD-L1 expression (69.6% vs. 34.0%, *P* = 0.0006, Table 2, Fig. 2a). Patients with larger tumor volume showed higher PD-L1 expression in IHC staining than those with smaller volume (Fig. 2b). We did not determine a relationship between tumor volume and CD8^+^ TIL levels (*P* = 0.171, Table 2, Fig. 2c–d). Moreover, patients with low CD8^+^ TIL levels showed a significantly higher rate of PTBE than those with high CD8^+^ TIL levels (57.1% vs. 18.2%, *P* = 0.0001, Table 2, Fig. 2e), whereas PD-L1 expression was not associated with PTBE (*P* = 0.442, Table 2, Fig. 2f). The number of CD8^+^ TILs in IHC staining was significantly higher in patients without PTBE than that in patients with PTBE (Fig. 2g). Age, gender, smoking, drinking, preoperative symptoms, and tumor sites were not associated with PD-L1 expression and CD8^+^ TIL levels. Moreover, as shown in Supplementary Fig. 1a and b, we did not determine the relationships among meningiomas grades, PD-L1 expression, and CD8^+^ TIL levels. Further, we performed regression analysis to eliminate the interference of confounding factors and determine the factors associated with the expression of the immune markers. Tumor volume was independently correlated with PD-L1 expression, which was also shown by univariate and multivariate logistic regression analyses (*P* = 0.003, HR = 5.288, 95%CI, 1.786–15.651; Table 3). Univariate logistic regression analysis showed that PTBE was significantly associated with CD8^+^ TIL levels (*P* < 0.001; Table 4). Multivariate logistic regression analysis showed that PTBE was an independent predictor of CD8^+^ TIL levels (*P* = 0.001, HR = 0.176, 95%CI, 0.065–0.477; Table 4), and no association was found between meningioma WHO grade and PD-L1 expression and CD8^+^ TIL levels (*P* = 0.648, *P* = 0.794, respectively; Table 3 and Table 4).

**Table 2:** Correlation of PD-L1 expression, CD8^+^ TIL levels and clinical features

Clinical features	PD-L1 expression			CD8^+^ TIL levels		
	Low	High	*P* value	Low	High	*P* value
Age (years)			0.755			0.751
≤ 46	24	23		24	23	
>46	22	24		25	21	
Gender			0.848			0.642
Male	10	11		12	9	
Female	36	36		37	35	
Smoking			0.797			0.491
No	40	40		41	39	
Yes	6	7		8	5	
Drinking			0.450			0.607
No	39	37		41	35	
Yes	7	10		8	9	
Preoperative symptoms						
Visual impairment			0.757			0.610
No	36	38		38	36	
Yes	10	9		11	8	
Headache			0.067			0.982
No	41	35		40	36	
Yes	5	12		9	8	
Limb weakness			0.450			0.272
No	39	37		38	38	
Yes	7	10		11	6	
Epilepsy			0.486			0.164
No	43	41		42	42	
Yes	3	6		7	2	
Tumor volume (cm^3^)			*0.0006*			0.171
≤ 42.89	32	16		22	26	
>42.89	14	31		27	18	
Peritumoral brain edema			0.442			*0.0001*
No	30	27		21	36	
Yes	16	20		28	8	
Tumor sites			0.558			0.496
Convexity	6	9		10	5	
Skull base	35	31		33	33	
Ventricle	5	7		6	6	
WHO grade			0.224			0.318
I	30	24		27	27	
II	7	14		14	7	
III	9	9		8	10	

The median value of PD-L1 and CD8^+^ TIL expression was used as cutoff points to divide the cohort into groups with low and high

**Table 3:** Univariate and multivariate logistic regression of clinical features for PD-L1 expression in meningioma

Variables	Univariate			Multivariate		
	HR	95% CI	*P* value	HR	95% CI	*P* value
Age (years)						
≤ 46	1			1		
>46	1.138	0.505–2.568	0.755	1.326	0.530–3.320	0.547
Gender						
Male	1			1		
Female	0.909	0.344–2.405	0.848	0.525	0.165–1.674	0.276
Tumor volume (cm^3^)						
≤ 42.89	1			1		
>42.89	4.429	1.854–10.580	*0.001*	5.288	1.786–15.651	*0.003*
PTBE						
No	1			1		
Yes	1.389	0.601–3.211	0.442	0.801	0.305–2.102	0.652
Tumor sites						
Convexity & ventricle	1			1		
Skull base	1.036	0.450–2.386	0.934	1.660	0.537–5.138	0.379
WHO grade						
I	1			1		
II-III	1.797	0.781–4.136	0.168	1.353	0.369–4.954	0.648

*PTBE* Peritumoral brain edema

**Table 4:** Univariate and multivariate logistic regression analyses of different parameters for CD8^+^ TIL levels in meningioma

Variables	Univariate			Multivariate		
	HR	95% CI	*P* value	HR	95% CI	*P* value
Age (years)						
≤ 46	1			1		
>46	0.877	0.388–1.980	0.751	1.037	0.414–2.597	0.939
Gender						
Male	1			1		
Female	1.261	0.473–3.361	0.643	1.361	0.407–4.560	0.617
Tumor volume (cm^3^)						
≤ 42.89	1			1		
>42.89	0.564	0.248–1.285	0.173	0.864	0.296–2.517	0.788
PTBE						
No	1			1		
Yes	0.167	0.064–0.432	*<0.001*	0.176	0.065–0.477	*0.001*
Tumor sites						
Convexity & ventricle	1			1		
Skull base	1.006	0.436–2.320	0.989	0.811	0.268–2.450	0.710
WHO grade						
I	1			1		
II-III	0.773	0.338–1.768	0.542	0.844	0.236–3.020	0.794

*PTBE* Peritumoral brain edema

### CD8^+^ TIL levels, PD-L1 expression, and tumor recurrence

**Fig. 3:**
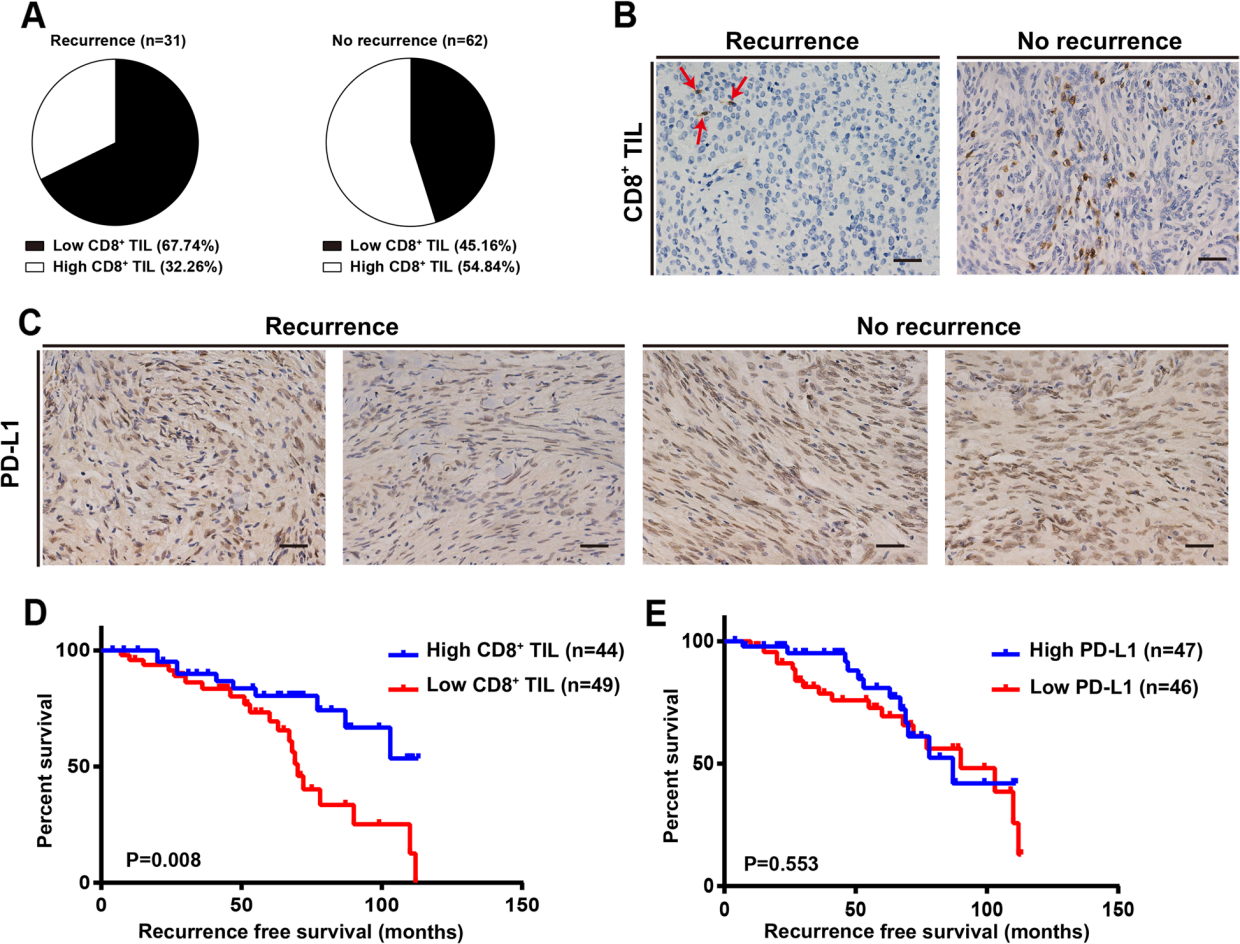
The association between immune markers and tumor recurrence. **a** The percentage of low levels of CD8^+^ TIL was higher in patients with recurrence than in the control patients without recurrence. **b** The number of CD8^+^ TILs in IHC staining was significantly less in patients with recurrence than in control patients without any recurrence. Scale bar = 20 μm. **c** The expression of PD-L1 in IHC staining was not significantly different between patients with and without recurrence. Kaplan–Meier analyses demonstrated that the CD8^+^ TIL levels (**d**) significantly correlated with recurrence-free survival, whereas the PD-L1 expression (**e**) did not. IHC, immunohistochemistry; PD-L1, programmed death ligand 1; TILs, tumor-infiltrating lymphocytes

We further determined the association between immune markers and tumor recurrence. Patients with recurrence and without recurrence (control) were matched with respect to age, gender, admission time, tumor sites, tumor volume, PTBE, Simpson grade resection, WHO grade, postoperative radiotherapy, and follow-up duration (Supplementary Table 2). Each patient was matched with 2 controls. The case group included 31 patients, whereas the control group included 62 patients; the average ages of the patients in the case and control groups were 46.39 ± 16.59 years (21–79 years) and 46.23 ± 16.33 years (22–79 years), respectively. The average tumor volumes were 45.24 ± 31.98 cm^3^ and 49.06 ± 41.64 cm^3^ for the cases and controls, respectively. Follow-up duration ranged from 7 to 112 months (53.58 ± 29.43 months) and 4 to 113 months (53.60 ± 29.05 months) for the cases and controls, respectively. Gender, tumor sites, PTBE, Simpson grade resection, WHO grade, and postoperative radiotherapy ratios of the cases and controls were exactly matched. The percentage of patients with low CD8^+^ TIL levels was higher in patients with recurrence than in control patients without recurrence (67.74% vs. 45.16%, *P* = 0.020; Fig. 3a, Table 5). The case-control OR was 0.325 (95%CI 0.125–0.840; Table 5) despite adjustments for the aforementioned factors. The number of CD8^+^ TILs in IHC staining was significantly less in patients with recurrence than in control patients without recurrence (Fig. 3b). The conditional logistic regression analysis showed no association between PD-L1 expression and tumor recurrence. PD-L1 expression in IHC staining showed no significant difference between patients with and without recurrence (Fig. 3c). Additionally, the Kaplan–Meier survival analysis indicated that lower CD8^+^ TIL levels were significantly associated with RFS in patients with meningioma (Fig. 3d). In contrast, the Kaplan–Meier survival curve and Cox regression analyses indicated no association between PD-L1 expression and RFS in patients with meningioma (Fig. 3e, Supplementary Table 3).

**Table 5:** Odds ratios for the immune markers for each group

Immune markers	Cases	Controls	*P* value	OR^a^	95% CI
CD8^+^ TIL levels					
low	21	28		1	
high	10	34	*0.020*	0.325	0.125–0.840
PD-L1 expression					
low	19	27		1	
high	12	35	0.054	0.378	0.141–1.015

^a^Odds ratio were adjusted for age, gender, admission time, tumor sites, tumor volume, peritumoral brain edema, Simpson grade resection, WHO grade, postoperative radiotherapy, and follow-up duration

## Discussion

A few reports are available on a relationship between tumor volume and PD-L1 expression concerning studies on meningioma. Studies have confirmed that PD-L1 promotes tumor growth in lung cancer [17] and prostate cancer [18]. In addition, a positive correlation between tumor volume and PD-L1 expression has been observed in renal cell carcinoma [19] and gastric cancer [20]. The present results suggested that PD-L1 may play a vital role in meningioma development, promoting tumor growth by decreasing the number of cytotoxic T lymphocytes and increasing the proportion of regulatory T cells [3, 4], resulting in increased tumor volume.

PTBE is a common complication of meningioma. Although the PTBE mechanism has not been clarified yet, recent studies indicate the involvement of intrinsic and anatomical factors [21]. High VEGF levels are present in meningioma with PTBE [22]; therefore, it is more prone to vasogenic brain edema, implying that a greater number of CD8^+^ TILs can infiltrate the TME with increased vascular permeability. However, we observed that PTBE was negatively associated with CD8^+^ TIL levels. Meningioma cells secrete VEGF [23], which induces angiogenesis [24] and promotes PTBE [22]. The VEGF gene overexpression decreases the levels of vascular cell adhesion molecules in endothelial cells, preventing the infiltration of CD8^+^ TILs into the TME [25]. Further, VEGF induces FasL upregulation in tumor endothelial cells, which induces T-cell apoptosis by binding to Fas expressed by activated T lymphocytes, leading to a decrease in CD8^+^ TILs in the TME [26]. Therefore, meningioma cells secrete VEGF that may promote PTBE and decrease the infiltration of the CD8^+^ TILs into the TME. We further analyzed the relationship between VEGF, CD8^+^ TILs, and PTBE in our samples to verify our assumption. As shown in Supplementary Fig. 2a and b, cases with high VEGF expression showed significantly lower CD8^+^ TILs levels than those with low VEGF levels (78.9% vs. 27.8%, *P* = 0.002). We found that cases with high VEGF levels showed a significantly higher rate of PTBE than those with low VEGF levels (63.2% vs. 16.7%, *P* = 0.004, Supplementary Fig. 2c).

Several studies have shown that PD-L1 is highly expressed in higher-grade meningiomas [27–30]. However, our study showed no significant correlation between them (*P* = 0.648, Table 3, Supplementary Fig. 1a), potentially because of the limited number of higher-grade meningiomas (*n* = 39, Table 1). The relationship between CD8^+^ TIL levels and the WHO grade of meningioma is not clear. We did not find an association (*P* = 0.794, Table 4, Supplementary Fig. 1b), which was consistent with the results of Rapp C [31]. However, several studies showed a decrease in the number of CD8^+^ TILs in higher-grade meningiomas [30, 32]. Therefore, the relationship between PD-L1 expression and CD8^+^ TIL levels and the WHO grade of meningioma is controversial and needs to be further explored.

The present results showed that low CD8^+^ TIL levels were associated with tumor recurrence in meningioma and increased tumor recurrence by 3 times (OR = 0.325, Table 5). These results were consistent with those of Rapp C [31], which showed that a higher number of cytotoxic TILs was associated with improved progression-free survival. In another study, high CD8^+^ TIL levels were associated with improved RFS in atypical meningioma [32]. An association between high CD8^+^ TIL levels and better RFS was also observed in NSCLC [33] and hepatocellular carcinoma [34]. Thus, CD8^+^ TILs may be a potential marker to predict meningioma recurrence, which should be investigated in further prospective research. The Kaplan–Meier survival curve and Cox regression analyses indicated that PD-L1 expression was not associated with meningioma recurrence (Fig. 3e, Supplementary Table 3), which was consistent with previous study results [28, 30]. However, one study discovered that high PD-L1 expression was an independent predictor of worse RFS in meningioma [27], and another study showed that PD-L1 expression was correlated with poor survival outcomes in meningioma [29]. Therefore, controversy exists in the field of meningioma regarding the association between PD-L1 expression and clinical outcomes. These controversial findings may be attributed to various reasons such as some samples with high PD-L1 expression did not contribute to tumor recurrence because PD-L1 was expressed on macrophages [29] rather than tumor cells (Supplementary Fig. 3). Further, intra- and inter-tumoral heterogeneities may cause a difference in PD-L1 expression. Therefore, the association between PD-L1 expression and meningioma recurrence needs further investigation.

Our findings contribute to limited studies available on PD-L1 expression and CD8^+^ TIL levels in the TME of meningioma. Our results showed that clinical features were associated with PD-L1 expression and CD8^+^ TIL levels in meningioma. A few studies on the relationship between clinical features and immune markers in meningioma are available. PD-L1 serves as a biomarker for predicting response to PD1/PD-L1 immunotherapy in other tumors types [35], and studies have shown that pre-existing CD8^+^ TILs are important for patients to benefit from PD1/PD-L1 immunotherapy [16]. Therefore, knowing that bigger tumor volumes are associated with PD-L1 expression could help choose patients that have more chances of benefitting from checkpoint blockade treatment. In contrast, patients with meningioma with PTBE are associated with lower CD8^+^ TIL levels, indicating that these patients may have difficulty benefiting from checkpoint blockade treatment. Moreover, we observed that CD8^+^ TIL levels were associated with meningioma recurrence. This finding may have clinical application in the postoperative management of meningioma, particularly in predicting tumor recurrence during routine follow-up. However, given the limitations of a case-control study, we cannot draw a causal relationship between CD8^+^ TIL levels and tumor recurrence. Therefore, prospective studies are needed to investigate the relationship between CD8^+^ TIL levels and tumor recurrence to establish CD8^+^ TIL levels as a predictive biomarker.

We performed individual matching, conditional logistic regression, and multivariate logistic regression analyses to control confounding bias and selection bias. The information bias was controlled by objective electronic data and standard methods of measuring the expression of immune markers. Though we attempted to control the biases in our study, it may have an inevitable potential selection bias because of a low recurrence rate in meningioma, resulting in a limited number of patients. Therefore, further studies with a large sample size are required to draw definitive conclusions.

## Conclusion

Tumor volume was associated with PD-L1 expression, and PTBE was an independent predictor of CD8^+^ TIL levels in meningioma. CD8^+^ TIL levels correlated with tumor recurrence in meningioma.

## Supplementary Information

**Additional file 1: Supplementary Table 1.**

**Additional file 2: Supplementary Table 2.**

**Additional file 3: Supplementary Table 3.**

**Additional file 4: Supplementary Fig 1.**

**Additional file 5: Supplementary Fig 2.**

**Additional file 6: Supplementary Fig 3.**

## Data Availability

The datasets generated and/or analyzed during the current study are not publicly available due their containing information that could compromise the privacy of research participants, but are available from the corresponding author on reasonable request.

## Abbreviations

PD-L1: Programmed death ligand 1
PD-1: Programmed cell death 1
NSCLC: Non-small-cell lung cancer
TME: Tumor microenvironment
CD8^+^ TILs: CD8^+^ tumor-infiltrating lymphocytes
IHC: Immunohistochemistry
PTBE: Peritumoral brain edema
MRI: Magnetic resonance imaging
RFS: Recurrence-free survival
ORs: Odds ratios
CI: Confidence interval
VEGF: Vascular endothelial growth factor
