# Supplementary material for: Programmed death ligand 1 and tumor-infiltrating CD8+ T lymphocytes are associated with the clinical features in meningioma
Source: BMC Cancer. 2022 Nov 12;22:1171. doi: 10.1186/s12885-022-10249-4 (PMC9655806; doi:10.1186/s12885-022-10249-4)
Supplement: Supplementary file 1 — Additional file 1: Supplementary Table 1. [file 12885_2022_10249_MOESM1_ESM.docx]

| **Supplementary Table 1.** The definition of Simpson Grade | |
| --- | --- |
| **Simpson Grade** | **Definition** |
| Grade I | complete removal including resection of underlying bone and associated dura |
| Grade II | complete removal and coagulation of dural attachment |
| Grade III | complete removal without resection of dura or coagulation |
| Grade IV | subtotal resection |
