# Supplementary material for: Programmed death ligand 1 and tumor-infiltrating CD8+ T lymphocytes are associated with the clinical features in meningioma
Source: BMC Cancer. 2022 Nov 12;22:1171. doi: 10.1186/s12885-022-10249-4 (PMC9655806; doi:10.1186/s12885-022-10249-4)
Supplement: Supplementary file 2 — Additional file 2: Supplementary Table 2. [file 12885_2022_10249_MOESM2_ESM.docx]

| **Supplementary Table 2 \|** Baseline characteristics of recurrence meningioma cases and controls. | | | | | | | |
| --- | --- | --- | --- | --- | --- | --- | --- |
|  | Cases (n=31) | | | Controls (n=62) | | |  |
| Variables | No. | % |  | No. | % |  | P value |
| Age (years） |  |  |  |  |  |  | 0.964 |
| Mean ± SD | 46.39±16.591 | |  | 46.23±16.329 | |  |  |
| Gender |  |  |  |  |  |  | 1.000 |
| Male | 7 | 22.6 |  | 14 | 22.6 |  |  |
| Female | 24 | 77.4 |  | 48 | 77.4 |  |  |
| Tumor sites |  |  |  |  |  |  | 1.000 |
| Convexity | 5 | 16.1 |  | 10 | 16.1 |  |  |
| Skull base | 22 | 71 |  | 44 | 71 |  |  |
| Ventricle | 4 | 12.9 |  | 8 | 12.9 |  |  |
| Tumor volume (cm^3^) |  |  |  |  |  |  | 0.655 |
| Mean ± SD | 45.24±31.98 | |  | 49.06±41.64 | |  |  |
| Peritumoral brain edema |  |  |  |  |  |  | 1.000 |
| No | 19 | 61.3 |  | 38 | 61.3 |  |  |
| Yes | 12 | 38.7 |  | 24 | 38.7 |  |  |
| Simpson grade |  |  |  |  |  |  | 1.000 |
| I | 9 | 29 |  | 18 | 29 |  |  |
| II | 7 | 22.6 |  | 14 | 22.6 |  |  |
| III | 11 | 35.5 |  | 22 | 35.5 |  |  |
| IV | 4 | 12.9 |  | 8 | 12.9 |  |  |
| WHO grade |  |  |  |  |  |  | 1.000 |
| I | 18 | 58.1 |  | 36 | 58.1 |  |  |
| II | 7 | 22.6 |  | 14 | 22.6 |  |  |
| III | 6 | 19.4 |  | 12 | 19.4 |  |  |
| Postoperative radiotherapy | |  |  |  |  |  | 1.000 |
| No | 26 | 83.9 |  | 52 | 83.9 |  |  |
| Yes | 5 | 16.1 |  | 10 | 16.1 |  |  |
| Follow-up duration (months） | |  |  |  |  |  | 0.998 |
| Mean ± SD | 53.58±29.43 | |  | 53.60±29.05 | |  |  |
