# Supplementary material for: Programmed death ligand 1 and tumor-infiltrating CD8+ T lymphocytes are associated with the clinical features in meningioma
Source: BMC Cancer. 2022 Nov 12;22:1171. doi: 10.1186/s12885-022-10249-4 (PMC9655806; doi:10.1186/s12885-022-10249-4)
Supplement: Supplementary file 3 — Additional file 3: Supplementary Table 3. [file 12885_2022_10249_MOESM3_ESM.docx]

| **Supplementary Table 3 \|** Univariate and multivariate analyses of different parameters for recurrence free survival of 93 meningiomas patients | | | | | | | | |
| --- | --- | --- | --- | --- | --- | --- | --- | --- |
| Variables |  | Univariate | | |  | Multivariate | | |
|  |  | HR | 95% CI | P value |  | HR | 95% CI | P value |
| CD8^+^ TIL levels | |  |  |  |  |  |  |  |
| Low |  | 1 |  |  |  | 1 |  |  |
| High |  | 0.372 | 0.174-0.795 | *0.011* |  | 0.247 | 0.085-0.714 | *0.010* |
| PD-L1 expression | |  |  |  |  |  |  |  |
| Low |  | 1 |  |  |  | 1 |  |  |
| High |  | 0.801 | 0.384-1.672 | 0.554 |  | 0.746 | 0.245-2.271 | 0.606 |
| Age (years) | |  |  |  |  |  |  |  |
| ≤46 |  | 1 |  |  |  | 1 |  |  |
| ＞46 |  | 1.065 | 0.525-2.161 | 0.862 |  | 1.177 | 0.400-3.466 | 0.768 |
| Gender |  |  |  |  |  |  |  |  |
| Male |  | 1 |  |  |  | 1 |  |  |
| Female |  | 0.550 | 0.232-1.303 | 0.174 |  | 0.668 | 0.125-3.578 | 0.637 |
| Tumor volume (cm^3^) | |  |  |  |  |  |  |  |
| ≤42.89 |  | 1 |  |  |  | 1 |  |  |
| ＞42.89 |  | 1.197 | 0.552-2.593 | 0.649 |  | 0.389 | 0.062-2.451 | 0.315 |
| Peritumoral brain edema | |  |  |  |  |  |  |  |
| No |  | 1 |  |  |  | 1 |  |  |
| Yes |  | 1.150 | 0.545-2.427 | 0.714 |  | 0.729 | 0.179-2.966 | 0.659 |
| Tumor sites | |  |  |  |  |  |  |  |
| Convexity & ventricle | | 1 |  |  |  | 1 |  |  |
| Skull base | | 0.606 | 0.284-1.295 | 0.196 |  | 0.787 | 0.188-3.295 | 0.743 |
| Simpson grade | |  |  |  |  |  |  |  |
| I-II |  | 1 |  |  |  | 1 |  |  |
| III-IV + postoperative radiotherapy | | 0.383 | 0.101-1.447 | 0.157 |  | 0.883 | 0.088-8.836 | 0.916 |
| WHO grade | |  |  |  |  |  |  |  |
| I |  | 1 |  |  |  | 1 |  |  |
| II-III |  | 1.349 | 0.631-2.884 | 0.439 |  | 5.964 | 0.478-74.432 | 0.166 |
