# Supplementary figures and images for: Programmed death ligand 1 and tumor-infiltrating CD8+ T lymphocytes are associated with the clinical features in meningioma
Source: BMC Cancer. 2022 Nov 12;22:1171. doi: 10.1186/s12885-022-10249-4 (PMC9655806; doi:10.1186/s12885-022-10249-4)

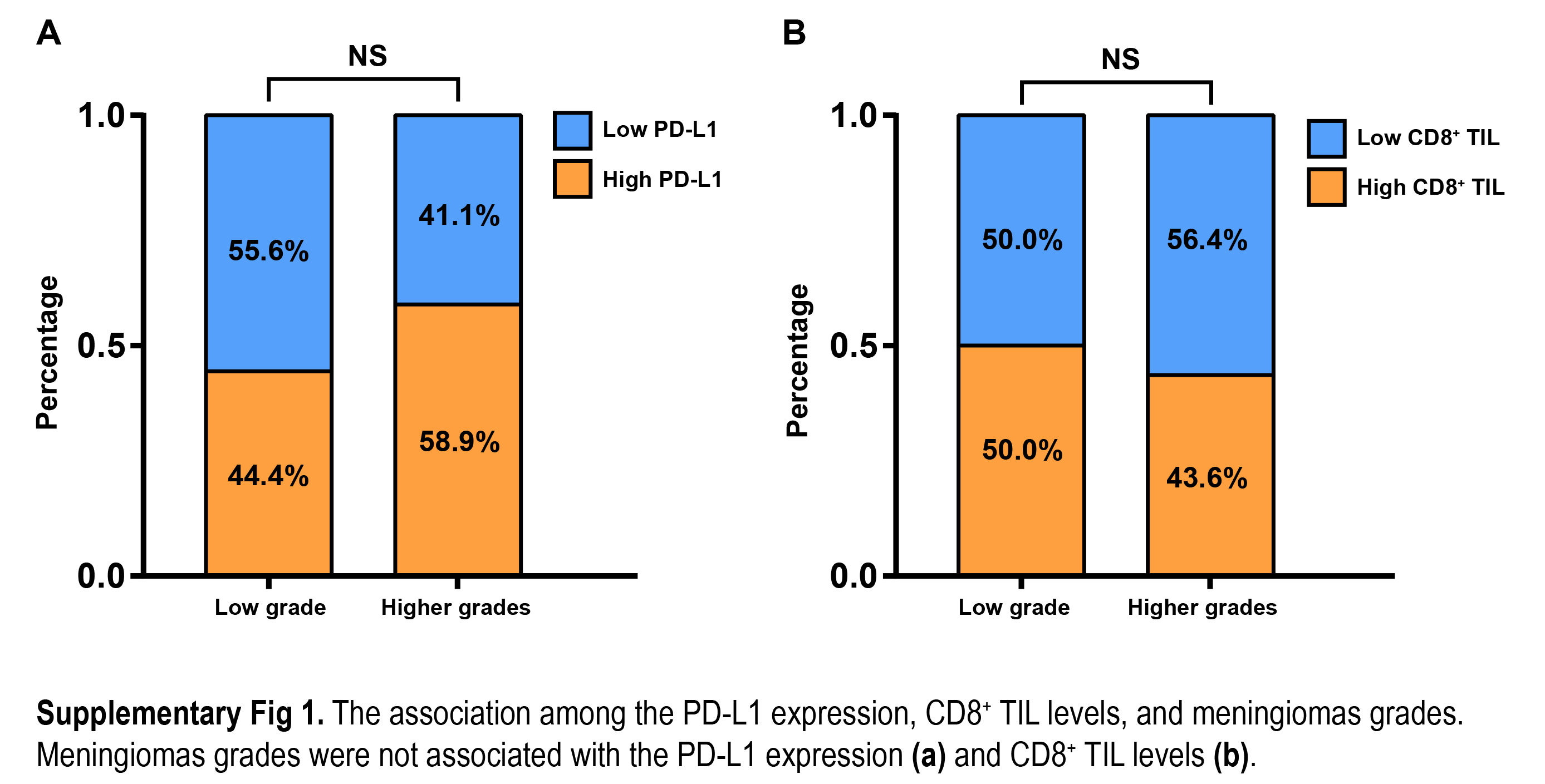

Supplement: Supplementary file 4 — Additional file 4: Supplementary Fig 1. [file 12885_2022_10249_MOESM4_ESM.tif]

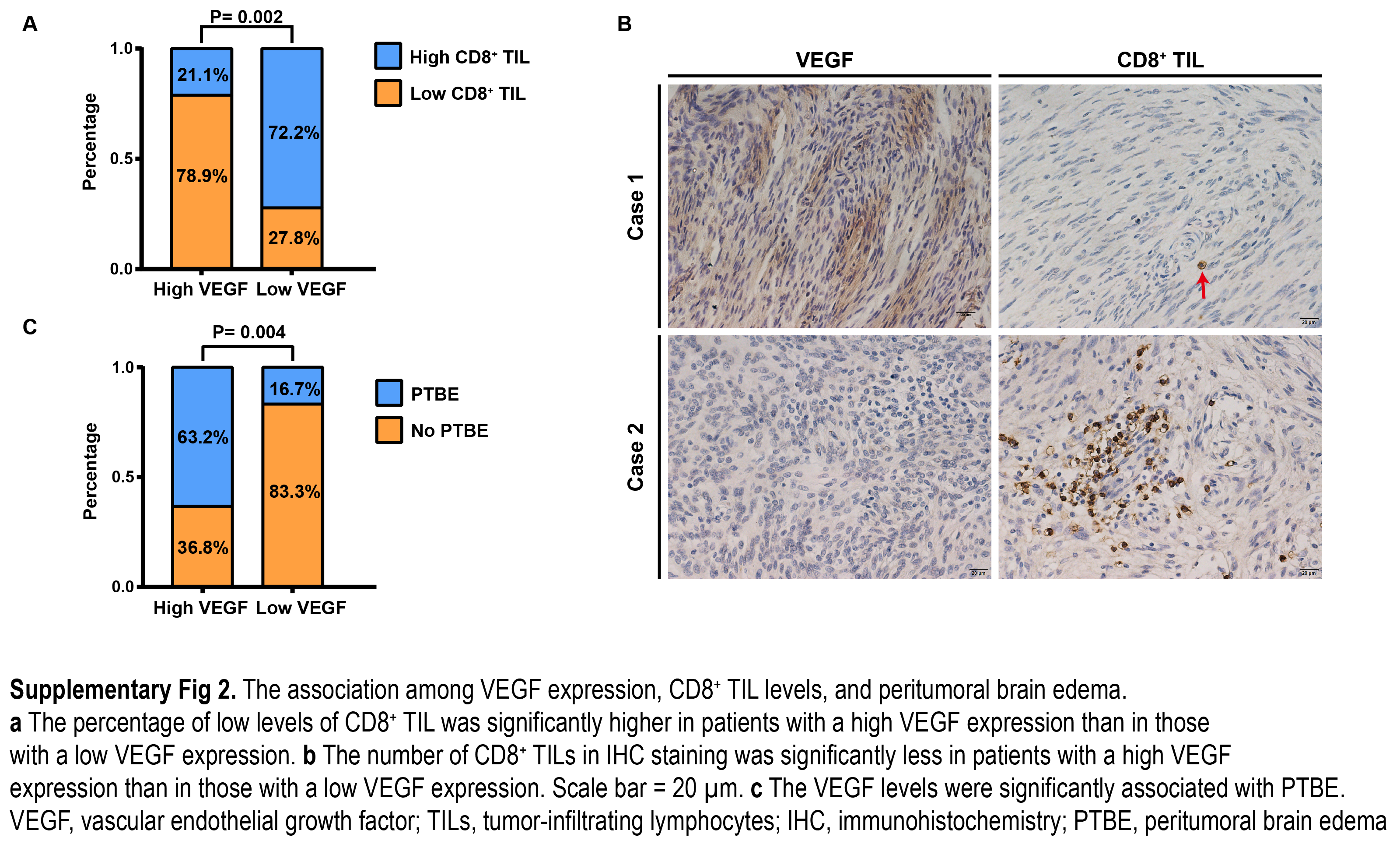

Supplement: Supplementary file 5 — Additional file 5: Supplementary Fig 2. [file 12885_2022_10249_MOESM5_ESM.tif]

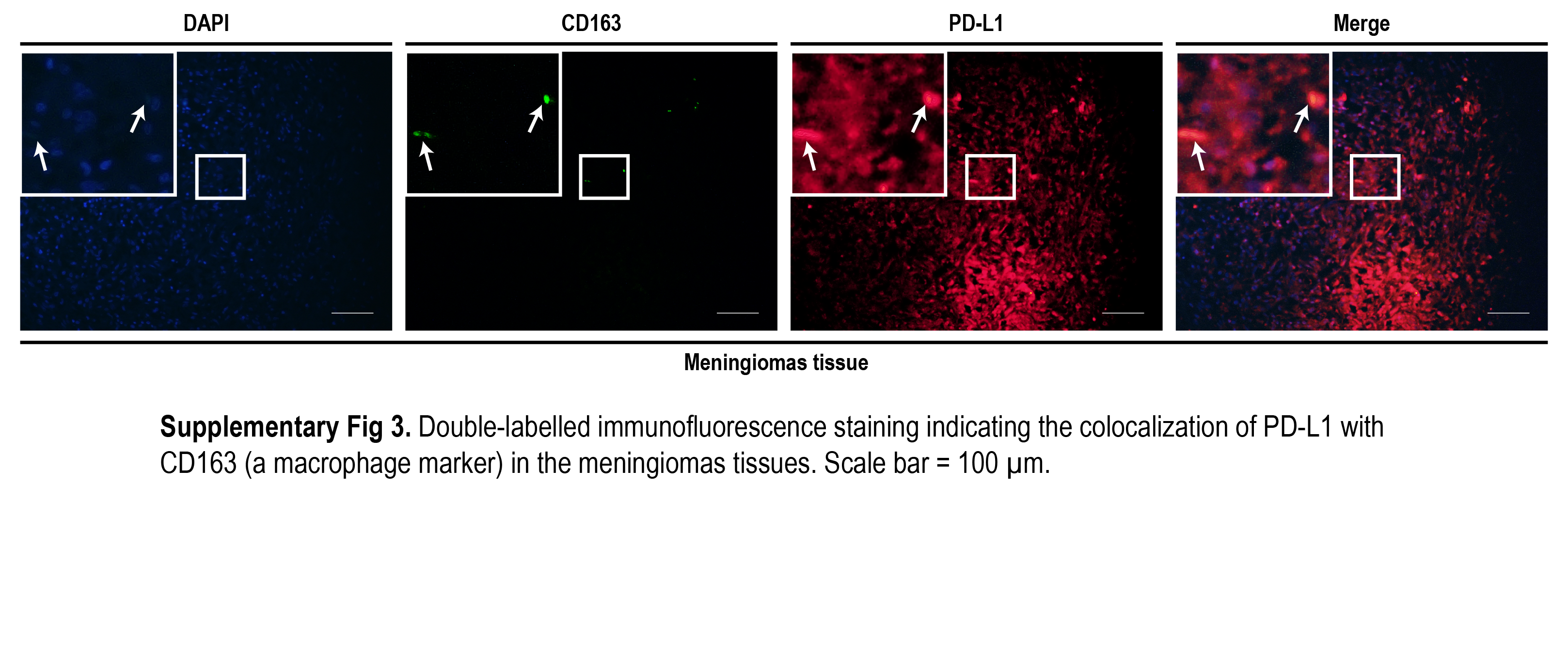

Supplement: Supplementary file 6 — Additional file 6: Supplementary Fig 3. [file 12885_2022_10249_MOESM6_ESM.tif]
